# MNDA expression and its value in differential diagnosis of B-cell non-Hodgkin lymphomas: a comprehensive analysis of a large series of 1293 cases

**DOI:** 10.1186/s13000-024-01481-6

**Published:** 2024-04-16

**Authors:** Li-Fen Zhang, Yan Zhang, Rou-Hong Shui, Hong-Fen Lu, Wen-Hua Jiang, Xu Cai, Xiao-Qiu Li, Bao-Hua Yu

**Affiliations:** 1 Department of Pathology, Fudan University Shanghai Cancer Center, Dong-an Road 270, Xuhui District, Shanghai, CN 200032 China; 2 Shanghai Medical College, Department of Oncology, Fudan University, Shanghai, CN 200032 China

**Keywords:** MNDA, Immunohistochemistry, Lymphoma, Differential diagnosis

## Abstract

**Aims:**

MNDA (myeloid nuclear differentiation antigen) has been considered as a potential diagnostic marker for marginal zone lymphoma (MZL), but its utility in distinguishing MZL from other B-cell non-Hodgkin lymphomas (B-NHLs) and its clinicopathologic relevance in diffuse large B-cell lymphoma (DLBCL) are ambiguous. We comprehensively investigated MNDA expression in a large series of B-NHLs and evaluated its diagnostic value.

**Methods:**

MNDA expression in a cohort of 1293 cases of B-NHLs and 338  cases of reactive lymphoid hyperplasia (RLH) was determined using immunohistochemistry and compared among different types of B-NHL. The clinicopathologic relevance of MNDA in DLBCL was investigated.

**Results:**

MNDA was highly expressed in MZLs (437/663, 65.9%), compared with the confined staining in marginal zone B-cells in RLH; whereas neoplastic cells with plasmacytic differentiation lost MNDA expression. MNDA expression was significantly higher in mantle cell lymphoma (MCL, 79.6%, *p* = 0.006), whereas lower in chronic lymphocytic leukemia/small lymphocytic lymphoma (CLL/SLL, 44.8%, *p* = 0.001) and lymphoplasmacytic lymphoma (LPL, 25%, *p* = 0.016), and dramatically lower in follicular lymphoma (FL, 5.2%, *p* < 0.001), compared with MZL. 29.6% (63/213) of DLBCLs were positive for MNDA. The cases in non-GCB group exhibited a higher rate of MNDA positivity (39.8%) compared to those in GCB group (16.3%) (*p* < 0.001), and MNDA staining was more frequently observed in DLBCLs with BCL2/MYC double-expression (50%) than those without BCL2/MYC double-expression (24.8%) (*p* = 0.001). Furthermore, there was a significant correlation between MNDA and CD5 expression in DLBCL (*p* = 0.036).

**Conclusions:**

MNDA was highly expressed in MZL with a potential utility in differential diagnosis between MZL and RLH as well as FL, whereas its value in distinguishing MZL from MCL, CLL/SLL is limited. In addition, MNDA expression in DLBCL was more frequently seen in the non-GCB group and the BCL2/MYC double-expression group, and demonstrated a correlation with CD5, which deserves further investigation. The clinical relevance of MNDA and its correlation with the prognosis of these lymphomas also warrant to be fully elucidated.

## Introduction

Myeloid nuclear differentiation antigen (MNDA) was initially detected in human promyelocytic cell lines [1, 2] and used as a marker for myeloid neoplasms [3, 4]. Subsequently, Miranda et al. identified a low level of MNDA expression in a population of normal mantle B lymphocytes and a subset of B-cell lymphomas [5]. Since being identified as a useful marker for the recognition of marginal zone lymphoma (MZL) by Kanellis et al. in 2009 [6], MNDA has been considered as a diagnostic marker for MZL recently in routine clinical practice [7]. MNDA showed a potential power in distinguishing nodal marginal zone lymphoma (NMZL) from follicular lymphoma (FL), given its high frequency in MZL and very limited expression in FL [6, 8]. However, a relatively higher expression of MNDA in FL was also reported by Wang et al. (21%) [9] and its frequency in FL of different grades is unclear. In addition, although MNDA expression has been found in various B-cell non-Hodgkin lymphomas (B-NHLs), studies on MNDA expression in B-NHLs other than MZL are limited and inconclusive. High expression of MNDA (78-82%) in mantle cell lymphoma (MCL) was demonstrated [6, 9], but a dramatically lower positivity (6.4%) was also reported by Metcalf et al [8]. Moreover, the frequency of MNDA expression in cases of chronic lymphocytic leukemia/small lymphocytic lymphoma (CLL/SLL, 12.9-76%), lymphoplasmacytic lymphoma (LPL, 27.7-83%) and diffuse large B-cell lymphoma (DLBCL, 3.3-45%) were even largely varied [6, 8–11]. Therefore, the expression of MNDA in diverse B-NHLs and its clinicopathological significance are needed to be further evaluated in larger study cohorts.

Herein, we comprehensively analyzed the expression of MNDA with the largest series of MZLs and other common types of small B-NHLs by far and evaluated its differential diagnostic value. The expression and clinical relevance of MNDA in DLBCL were also investigated in our study.

## Materials and methods

### Case Selection

A total of 1293 cases with various subtypes of B-NHL and 338 cases with reactive lymphoid hyperplasia (RLH) were included, which were diagnosed between September 2018 to January 2021 in the Department of Pathology, Fudan University Shanghai Cancer Center. All cases were reviewed by 2 experienced pathologists according to the World Health Organization (WHO) classification of tumors of hematopoietic and lymphoid tissues [12, 13].

### Immunohistochemistry (IHC) and in situ hybridization (ISH) for Epstein-Barr virus encoded small RNA (EBER)

The corresponding paraffin-embedded tissue sections were used for a routine hematoxylin and eosin (H&E) stain, immunohistochemical procedure, and detection of EBER. IHC was performed by an automatic immune-stainer (Ventana Medical System Inc., Roche Tuson, AZ, USA). The primary antibodies included MNDA (clone 253 A, Abcam, Cambridge, UK, 1:600 dilution); CD20, BCL2, CD3, CD5, CD30, Ki67, CyclinD1 (Roche, AZ, USA); CD10, CD21 (Maixin, Fuzhou, China); BCL6 (Leica, Germany); MYC (Abcam, Cambridge, UK); MUM1, , CD23 (Dako, CPH, DK). Based on previously described scoring methods with a modification, MNDA expression was scored according to the intensity of nuclear staining (0, negative; 1, weak; 2, moderate; 3, strong staining) and the percentage of positive tumor cells (0, less than 10%; 1, 10-25%; 2, 26-50%; 3, 51-75%; 4, 76-100% positively stained tumor cells). The final score was obtained by multiplying these two scores and a final score of ≥ 3 was defined as positive [5]. Histocytes and granulocytes with strong positivity served as the internal control.

DLBCL cases were classified into germinal center B-cell (GCB) and non-GCB subtypes according to the Hans algorithm for cell-of-origin (COO) classification [14]. BCL2/ MYC double-expression refers to those cases with ≥ 50% BCL2 and ≥ 40% MYC positivity according to the WHO classification [12, 13].

The fluorescein-labeled oligonucleotide probe (Leica, Germany) was designed for ISH to detect the status of EBER in DLBCL cases.

### Statistics

All the statistical analysis was conducted by SPSS 25.0 (SPSS Inc., Chicago IL, USA). The comparison of MNDA expression among different lymphomas and its correlation with clinicopathological parameters in DLBCL were analyzed by χ2 analysis and Spearman test. Statistical significance was defined as *p* < 0.05.

## Results

### MNDA expression in RLH cases

**Fig. 1:**
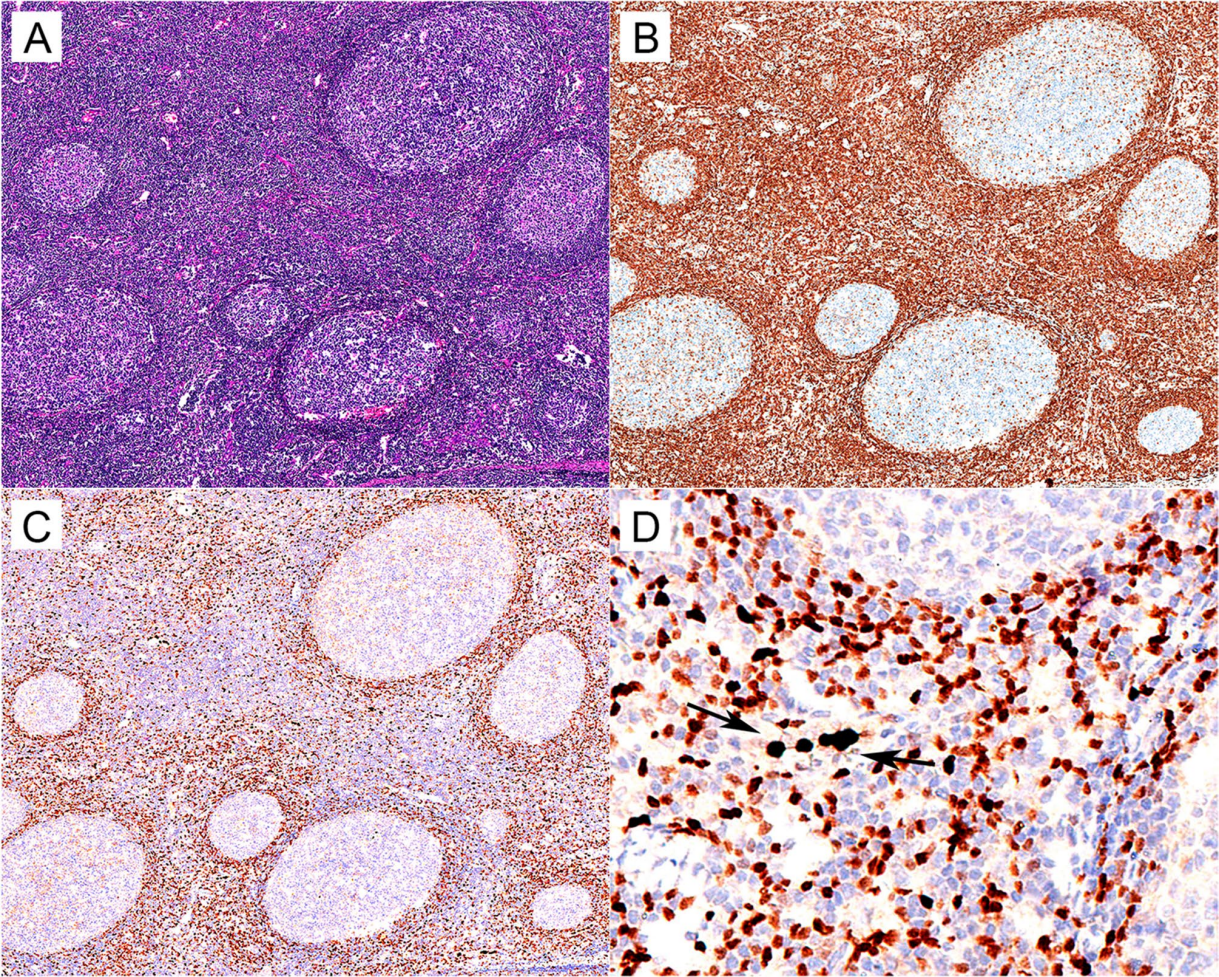
MNDA expression in RLH case. (**A**) H&E (×50). (**B**) BCL-2 (×50). (**C**) Nuclear MNDA staining is mostly located in the B lymphocytes of the marginal/mantle zone, with moderate reactivity. There is no strong staining of MNDA in B lymphocytes within the germinal center (×50). (**D**) Magnification for the marginal/mantle zone and inter-follicular areas (×400). The arrow emphasizes the strong staining of MNDA in histocytes and granulocytes

As a control, 338 RLH cases were enrolled in the current study, including 98 cases from lymph nodes and 240 cases from extra-nodal sites including the tonsil, stomach, intestine, orbit, lung, nasopharynx, salivary gland, spleen, thymus, bladder, and testis. In most cases, IHC staining revealed moderate reactivity for MNDA mainly in B lymphocytes located in the marginal/mantle zone of reactive follicles and occasionally in the lymphocytes within inter-follicular areas, whereas there was no strong staining of MNDA in B-cells within germinal centers. Notably, Histocytes and granulocytes in inter-follicular areas demonstrated strong positive staining of MNDA, which might serve as a distinct internal control (Fig. 1).

### MNDA expression in MZL

**Fig. 2:**
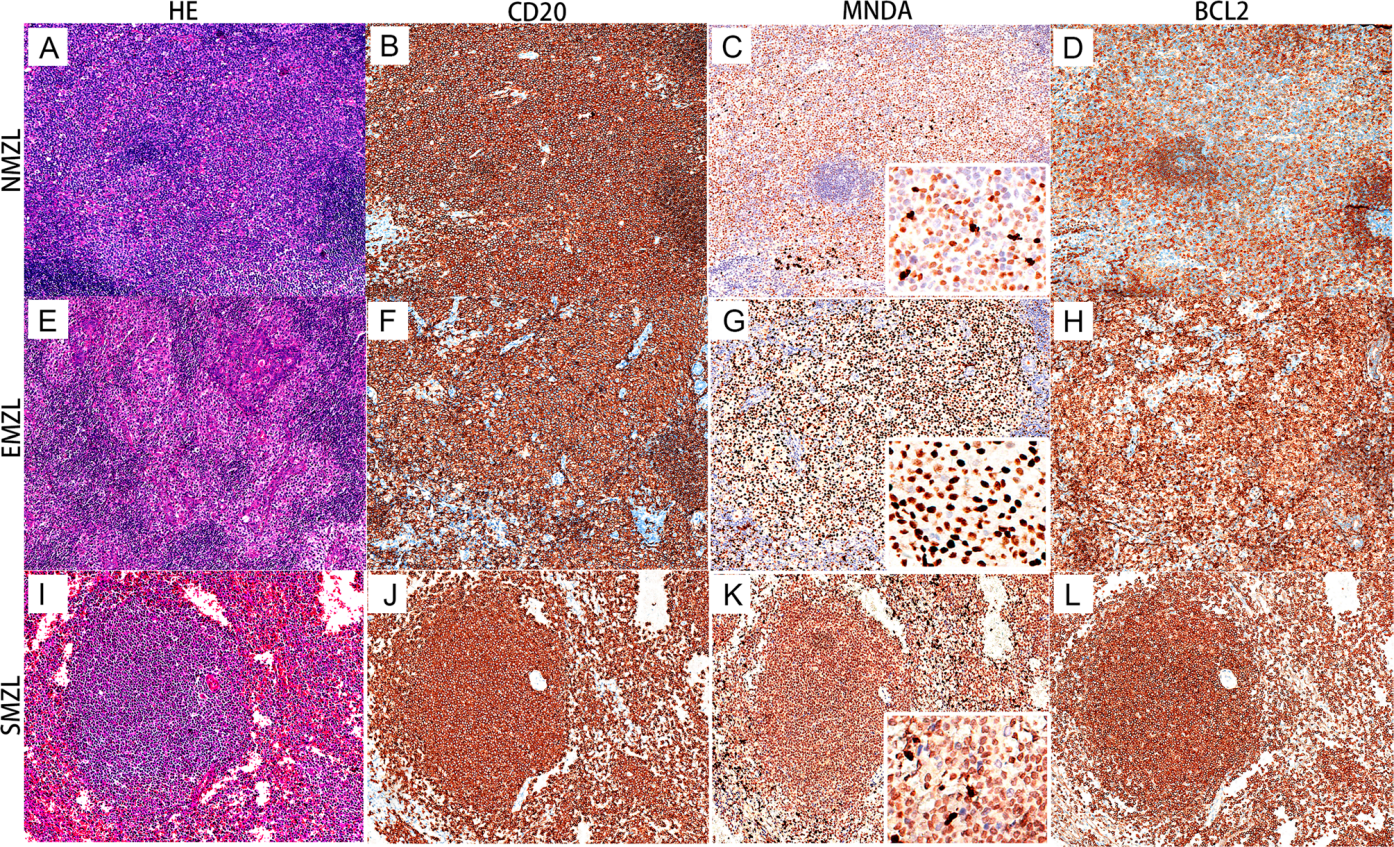
MNDA expression in 3 subtypes of MZL cases. (**A-D**) A case of NMZL in the cervical lymph node (×100). The neoplastic B cells diffusely expresse CD20, MNDA, and BCL2. The surviving germinal centers have no expression of MNDA and BCL2. (**E-H**) A case with EMZL from the salivary gland (×100). CD20, MNDA, and BCL2 present a pattern of diffuse positivity. (**I-L**) A case of SMZL (×100). MNDA marks the neoplastic cells in lymphoid follicles and infiltration in red pulp

MNDA expression in 1293 cases of B-NHL were summarized in Table 1. MNDA expression was found in 65.9% (437/663) of the cases with MZL, and MNDA staining was diffusely positive in all 3 subtypes of MZL with a moderate intensity (shown in Fig. 2). There was no significant difference in the frequency of MNDA expression between extranodal marginal zone lymphoma of mucosa-associated lymphoid tissue (MALT lymphoma/EMZL) (370/564, 65.6%) and NMZL (62/94, 66.0%) (*p* = 0.947). In addition, all 5 (100%) cases with splenic marginal zone lymphoma (SMZL) diffusely expressed MNDA (Fig. 2). Notably, MNDA expression in MZLs with plasmacytic differentiation (18/51, 35.3%) was significantly lower than those without plasmacytic differentiation (352/513, 68.6%) (*p* < 0.001). In cases with marked plasmacytic differentiation, MNDA staining, if any, was observed only in the neoplastic lymphocytes, whereas tumor cells lost MNDA when they acquired plasma cell morphology. Furthermore, MNDA staining was absent in those cases with almost complete plasmacytic differentiation, with only the few surviving follicles showing MNDA positive in marginal zone.

**Table 1:** Summary of MNDA expression in B-cell non-Hodgkin lymphoma

MNDA expression						
Lymphomas	Kanellis G, et al. (15%)	Metcalf R, et al. (15%)	Wang Z, et al. (20%)	Kivrak H, et al. (15%)	Righi S, et al. (NA)	Li-Fen Z, et al.
		grade1-2: 3/69(4.3%) grade 3: 3/41(7.3%)				grade 1–2: 5/162(3.1%)
FL	9/184(5%)		3/14(21%)	1/7(14%)	0/79(0%)	grade 3: 7/65(10.8%)
						Total:13/248(5.2%)
NZML	43/57(75%)	16/24(66.7%)	12/22(54%)	11/14(78%)	70/110(64%)	62/94(66.0%)
EMZL	19/20(95%)	27/44(61.4%)	21/31(68%)	11/17(64.7%)		370/564(65.6%)
SMZL	20/20(100%)	5/21(23.8%)	18/26(69%)	38/55(69%)	21/31(68%)	5/5(100%)
MCL	61/74(82%)	9/140(6.4%)	7/9(78%)	NA	27/61(44%,17BMB)	82/103(79.6%)
CLL/SLL	23/35(65%)	4/31(12.9%)	8/15(53%)	16/21(76%)	24/87(28%,17BMB)	26/58(44.8%)
LPL	10/12(83%)	2/8(25%)	3/8(37%)	5/18(27.7%)	3/10(30%)	2/8(25.0%)
DLBCL	34/75(45%)	2/61(3.3%)	NA	NA	NA	63/213(29.6%)
Total	477	439	125	132	378	1293

*Note* The thresholds of MNDA used by the investigators are indicated in parentheses on the second row of the table

*Abbreviation* NA, not available; MNDA, myeloid differentiation antigen; FL, follicular lymphoma; NMZL, nodal marginal zone lymphoma; EMZL, extranodal MZLs of mucosa-associated lymphoid tissue; SMZL, splenic marginal zone lymphoma; MZL, mantle cell lymphoma; CLL/SLL, chronic lymphocytic leukemia/small lymphocytic lymphoma; LPL, lymphoplasmacytic lymphoma; DLBCL, large B-cell lymphoma; BMB, bone marrow biopsy

### MNDA expression in other small B-NHLs

**Fig. 3:**
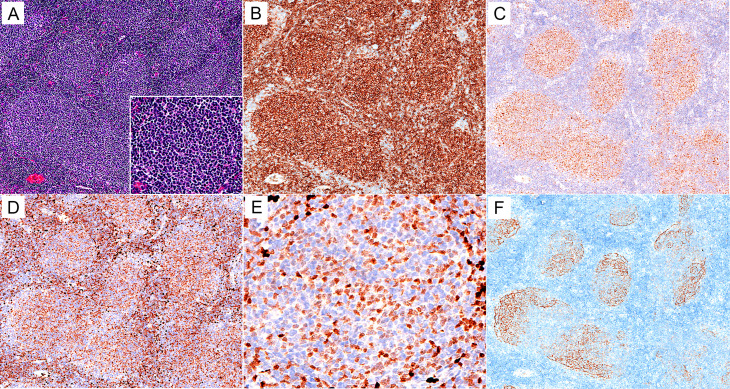
MNDA expression in a case of grade 1 FL. (**A**) H&E (×100). (**B, C**) Neoplastic cells are positive for CD10 (**B**, ×100) and BCL6 (**C**, ×100). (**D, E**) MNDA is diffusely expressed in neoplastic follicles, with scattered positivity in interfollicular areas (**D**, ×100; **E**,×400). (**F**) CD21 marks the neoplastic follicles (×100)

As for FL, in total, only 13 of 248 (5.2%) cases were positive for MNDA in our series, which was significantly lower than that in MZL cases (*p* < 0.001). We further investigated the MNDA expression in FLs of different grades. Except for 12 FLs of specific subtypes (including 7 paediatric-type FLs and 5 duodenal-type FLs) for which the grading was not used and 9 cases presenting challenges in grading due to limited tissue through needle biopsy, the remaining 227 FL cases were successfully graded. MNDA staining was observed in 3.1% (5/162) of grade 1 ∼ 2 FLs and 10.8% (7/65) of grade 3 ones. Morphologically, MNDA staining was diffusely and uniformly distributed in follicles, with scattered staining in inter-follicular areas (Fig. 3).

**Fig. 4:**
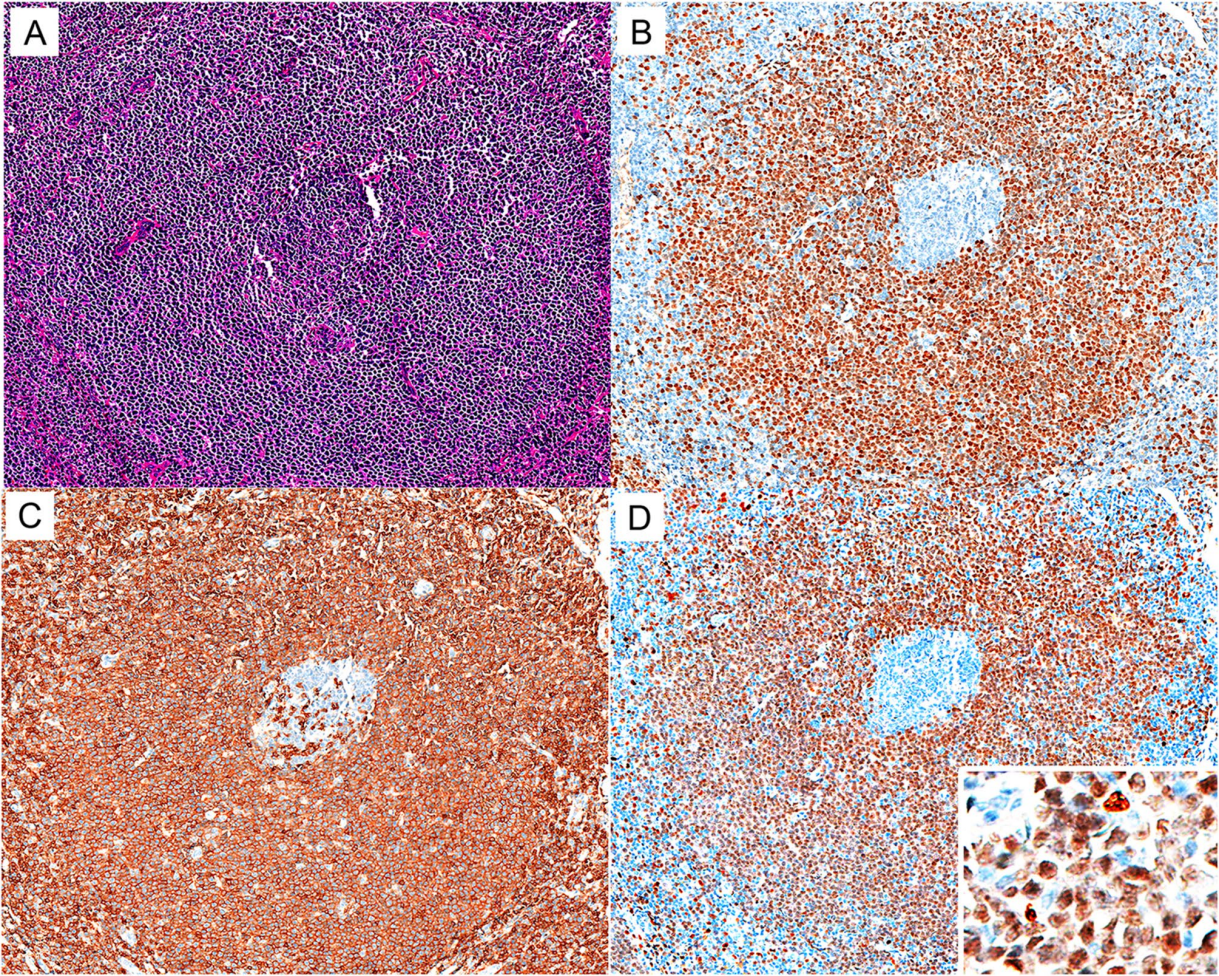
MNDA expression in MCL. (**A**) H&E (×100). (**B-D**) CyclinD1 (**B**), CD5 (**C**), and MNDA (**D**) are diffusely and uniformly positive in neoplastic cells (×100)

Worthy to be noticed, MNDA was diffusely expressed in 82 of 103 (79.6%) MCL cases (Fig. 4), which was significantly higher than that in MZL (*p* = 0.006). Among these, 2 of 3 cases of pleomorphic variant also demonstrated positive staining for MNDA with diffuse pattern.

**Fig. 5:**
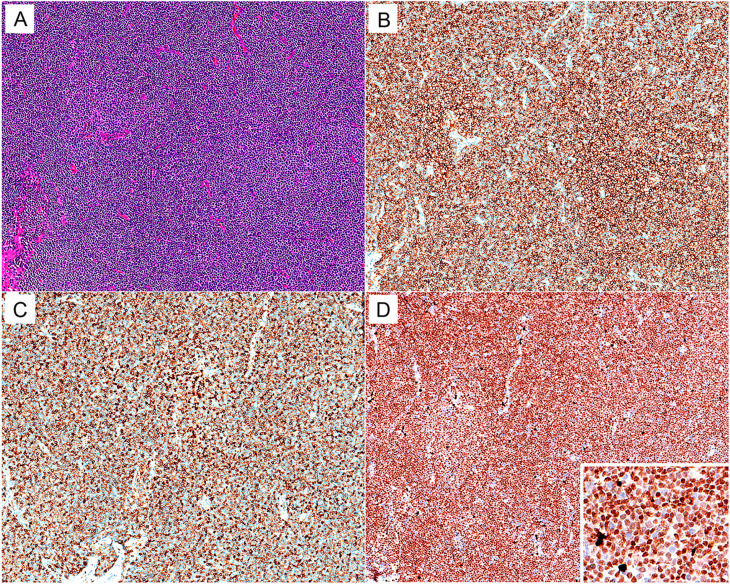
MNDA expression in CLL/SLL. (**A**) H&E (×100). A case of CLL/SLL from the cervical lymph node with a vaguely nodular appearance. (**B-D**) The neoplastic cells diffusely express CD23(**B**), CD5(**C**), and MNDA(**D**) (×100)

MNDA-positive staining was found in 44.8% (26/58) of CLL/SLL cases in our series, statistically lower than that in MZL (*p* = 0.001), and the staining was consistently diffuse and uniform (Fig. 5). In addition, 25% (2/8) of LPL cases were positive for MNDA in this study cohort.

### MNDA expression in DLBCL

**Fig. 6:**
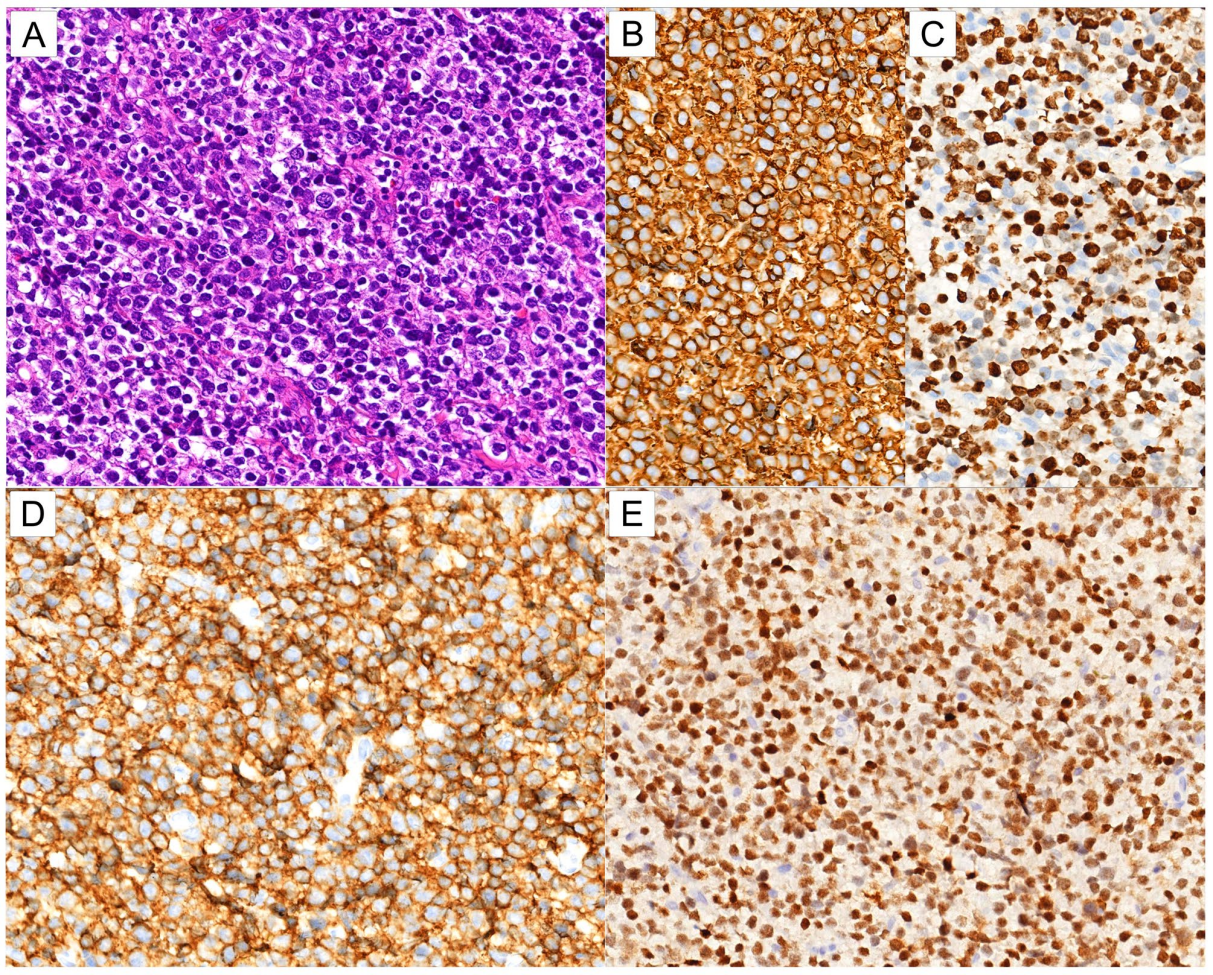
MNDA expression in DLBCL. (**A**) H&E (×400). (**B, C**) CD20(**B**, ×400), Ki67 (**C**,×400).(**D, E**) The tumor cells are diffusely positive for CD5(**D**, ×400) and MNDA (**E**, ×400)

The expression of MNDA and its clinicopathological significance in DLBCL were also investigated (Fig. 6). The results revealed that 63 of 213 (29.6%) DLBCL cases showed moderate reaction with MNDA. Positive staining of MNDA was more common in non-GCB group (45/113, 39.8%) than that in GCB group (14/86, 16.3%) (*p* < 0.001), and cases with BCL2/MYC double-expression (28/56, 50.0%) demonstrated more frequent MNDA staining than their counterparts (27/109, 24.8%) (*p* = 0.001). Interestingly, MNDA expression was more frequently observed in CD5-positive DLBCLs (9/17, 52.9%) than in CD5-negative ones (50/176, 28.4%) (*p* = 0.036). While, the expression of MYC, CD30, and EBER status showed no correlation with MNDA (Table 2).

**Table 2:** The correlation between MNDA expression and important clinicopathological parameters in DLBCL

Parameter	Total cases (*N* = 213)	MNDA expression(%)	p-value	
**Immunotype**			<0.001	
Non-GCB	113	45(39.8)		
GCB	86	14(16.3)		
ND	14			
**Gender**			0.057	
Female	116	28(24.1)		
Male	97	35(36.1)		
**Location**			0.179	
Nodal	36	14(38.9)		
Extra-nodal	177	49(27.7)		
**EBER**			0.290	
Positive	17	4(23.5)		
Negative	123	45(36.6)		
ND	73			
**BCL2**			0.004	
Positive	122	46(37.7)		
Negative	85	16(18.8)		
ND	6			
**MYC**			0.094	
Positive	94	36(38.3)		
Negative	73	19(26.0)		
ND	46			
**BCL2/MYC DE**			0.001	
Positive	56	28(50.0)		
Negative	109	27(24.8)		
ND	48			
**CD5**			0.036	
Positive	17	9(52.9)		
Negative	176	50(28.4)		
ND	20			
**CD30**			0.568	
Positive	16	5(31.2)		
Negative	72	28(38.9)		
ND	125			

*Abbreviation* ND, not done; DE, double expression; MNDA, myeloid differentiation antigen; DLBCL, diffuse large B-cell lymphoma; GCB, germinal center B-cell

## Discussion

MNDA has been found to be expressed in a large proportion of MZLs and the existing literature has suggested MNDA as a useful marker for identifying MZL [6, 8]. However, large series of studies on the expression of MNDA in various subtypes of B-NHL are lacking. Therefore, its potential utility in the differential diagnosis between MZL and other B-NHLs remains poorly understood and inconclusive, and its clinical relevance in DLBCL has never been elucidated by far. In this study, we thoroughly investigated the MDNA expression in a large cohort of diverse B-NHLs as well as RLH cases, which appeared to be the largest case series by far to the best of our knowledge.

Our results revealed frequent expression of MNDA in MZL, including both NMZL and EMZL, which was in line with some previous reports [8, 9], while slightly lower than the data from Kanellis et al. (75% and 95%) [6]. Consistent with the results from Kanellis et al. (100%) [6], MNDA was expressed in all of the SMZL cases in our series, although the sample was quite limited. MNDA staining in tumor cells of MZL cases is usually diffusely distributed, at least partially, which was different from that in RLH cases, mostly showing restricted staining to the mantle/marginal zone of lymphoid follicles. Therefore, MNDA might play an important role in the differential diagnosis of MZL and RLH. However, we noticed that MNDA positivity was less commonly observed in the MZL cases with considerable plasmacytic differentiation. In detail, while the lymphoid component exhibited a certain degree of MNDA expression, the neoplastic cells with plasmacytic differentiation lost MNDA. Our finding indicated limited value of MNDA in the recognization of MZL with extensive plasmacytic differentiation, which had never been documented in the previous literature. Moreover, the histocytes/granulocytes strongly positive for MNDA in the background should not be interpreted as neoplastic cells in clinical practice, which should be kept in mind to avoid mistakes.

Extremely low frequency of MNDA expression in FL cases of our series was consistent with the results of Kanellis et al. (5%) [6], but lower than those of Kivrak et al. (14%) and Wang et al. (21%) [9, 10]. Moreover, whereas MNDA expression was slightly higher in grade 3 FL, it was rarely seen in grade 1 ∼ 2 FL cases (3.1%), which was in accord with the results of Metcalf et al. (4.3%). Notably, the expression of MNDA in documented FL cases is mostly restricted to the thin perifollicular areas showing marginal zone differentiation and only in a few scattered centrocyte-like cells in the follicles [6, 8]. In contrast with the previous reports, MNDA-positive FL cases in our series demonstrated diffuse MNDA staining in neoplastic follicles and scattered in tumor cells in the inter-follicular areas [6, 8].

In clinical practice, distinguishing between MZL and FL is usually straightforward, given their different cell origin and growth patterns as well as immunophenotypes. In some situations, however, MZLs might present a nodular growth pattern or occasional CD10 aberrant expression, mimicking low-grade FL or sometimes low-grade FL might demonstrate a diffuse pattern, posing challenges for differential diagnosis between the two diseases. Our results in the current study further confirmed the efficacy of MNDA protein in distinguishing MZL from FL, which was in accord with the previous studies [6, 8]. However, Verdanet et al. demonstrated a similar frequency of MNDA expression in cutaneous MZL (4/13, 30.8%) and cutaneous FL (3/29,10.3%) (*p* = 0.18), which made the effectiveness of MNDA in the differential diagnosis of these two unusual lymphoma variants ambiguous [15].

Whereas Metcalf et al. showed a strikingly low expression of MNDA in MCL (6.4%) [8], our results revealed a high frequency (79.6%), similar to the majority of studies (from 78 to 82%) [6, 9]. Given a comparable or even more frequent MNDA expression in MCL compared with MZL, it is useless for MNDA in the distinguishment of these two types of B-NHL. The predilection of MNDA staining in the marginal zone/mantle B-cells and corresponding lymphomas warranted to be investigated further.

MNDA expression in CLL/SLL in the literature varied widely, ranging from 12.9 to 76% [6, 8–10], and we got a rate of 44.8% in the current study. The differences among these results might be partially due to the limited number of cases and the criteria for MNDA positivity varied across each study. For the reason that the thresholds utilized in the existing literature presented minimal differences, with no mention of positive intensity. We have modified the scoring methods based on previous studies and adopted a semi-quantitative method that considered both the positive intensity and the percentage of positive tumor cells. Although the frequency of MNDA in CLL/SLL is statistically lower than that in MZL, it seems that its capability in the differentiation between these two entities is limited in routine surgical pathology practice, given its non-negligible expression in nearly half of CLL/SLL cases. Therefore, a panel of IHC markers is still necessary for their differential diagnosis.

MNDA expression in LPL was 25-83% according to the existing literature [6, 8–11] and 25% in the current study, all with a small number of samples. Recently, Righi et al. showed a much lower frequency in LPLs (4% in the test set and 20% in the validation set) compared with 88% in SMZLs in bone marrow specimens [11], indicating that MNDA might be helpful in the differentiation between LPL and SMZL, especially when the status of *MYD88* L265P mutation is unavailable. Given the low incidence of LPL and the widely varied frequencies of reported MNDA expression in LPL, more cases need to be accumulated to further explore the features of MNDA expression in this rare type of B-NHL.

Studies focusing on MNDA expression in DLBCL are lacking, and our cohort represented the largest study series investigating MNDA in DLBCL as we know. Our results showed a frequency of 29.6% in DLBCL, which was lower than the data from Kanellis et al. (34/75, 45%) [6] whereas much higher than that from Metcalf et al. (2/61, 3.3%) [8]. For the first time, we further investigated the clinicopathological relevance of MNDA in DLBCL, and our results revealed a higher frequency of MNDA in non-GCB subtype and in BCL2/MYC double-expression group. Interestingly, MNDA had a significant correlation with CD5 expression in DLBCL in the current series, which had never been documented in the literature. As we mentioned above, MNDA tended to be positive in the majority of MCLs and a considerable number of CLL/SLL cases, in both of which CD5 is consistently positive as a specific diagnostic marker [16]. These observations suggested that aberrant MNDA overexpression might correlate with CD5 in B-NHLs, which is worthy of further study and the underlying mechanisms deserve further investigation.

Furthermore, the studies focused on the prognostic value of MNDA are limited, only a few in vitro studies suggested the prognostic potential of MNDA in CLL. For instance, Joshi et al. demonstrated that the expression of MNDA in CLL showed an inverse correlation with disease progression [17] and Bottardi et al. further revealed the potential role of MNDA in tumor suppression by regulating the apoptosis of neoplastic cells in CLL [18]. Unfortunately, more than 90% of the small B-NHLs in the current study were diagnosed within 5 years, the time is insufficient to achieve the expected target events necessary for survival analysis if prognostic follow-up has been conducted at this stage. Moreover, the number of MNDA-positive DLBCL cases for prognostic analysis is inadequate. Therefore, we will accumulate more cases and ensure a sufficient follow-up period for analyzing the prognostic value of MNDA in these lymphomas.

In summary, MNDA is highly expressed in MZL except those tumor cells with plasmacytic differentiation. MNDA might be an effective indicator for differentiating MZL from RLH and FL in clinical practice, but cannot be used to identify MZL from MCL and its value for distinguishing MZL from CLL/SLL is limited, thus a panel of markers is always indispensable in these circumstances. In DLBCL, the expression of MNDA has a correlation with immunophenotype and BCL2/MYC double-expression as well as CD5 expression.

## Data Availability

No datasets were generated or analysed during the current study.
